# Bullying in Fly-In-Fly-Out employees in the Australian resources sector: A cross-sectional study

**DOI:** 10.1371/journal.pone.0229970

**Published:** 2020-03-24

**Authors:** Peta Miller, Libby Brook, Norman Stomski, Graeme Ditchburn, Paul Morrison

**Affiliations:** College of Science, Health, Engineering and Education, Murdoch University, Murdoch, WA, Australia; Universitat de Valencia, SPAIN

## Abstract

**Background:**

Workplace bullying has diverse consequences at both the organisational and individual level. Anecdotal reports indicate that workplace bullying is an issue of particular concern for Australian FIFO workers, which may impact on psychosocial distress. However, no prior studies have examined this issue empirically in a FIFO worker cohort.

**Methods and materials:**

A cross-sectional survey study design was used to establish the prevalence of bullying in Australian FIFO, antecedents of bullying, and its association with psychosocial distress. Responses were received from 580 FIFO workers in the Australian resources sector. Primary outcome measures were Negative Acts Questionnaire-Revised, Beck Depression Inventory II, and Beck Hopelessness Scale. Logistic regression models were constructed to examine the association between bullying, suicide risk, and clinical depression.

**Results:**

Over half of the respondents experienced workplace bullying (55.7%), and about one-third reported moderate or more severe depression (32.3%). Being above the median age (OR = 0.51; 95% CI = 0.31–0.83) and having a supervisor who failed to promote collaboration (OR = 3.04; 95% CI = 1.84–5.04) were both significantly associated with experiencing bullying. Bullying was associated with an almost threefold increase in the likelihood of participants reporting increased suicide risk (OR = 2.70; 95% CI = 1.53–4.76). Bullying was also associated with participants being almost two and a half times more likely to report clinical depression (OR = 2.38; 95% CI = 1.40–4.05).

**Conclusion:**

The incidence of bullying in Australian FIFO workers has reached alarming proportions. Bullying was significantly associated with higher levels of clinical depression and suicide risk. The results highlight the need to implement in the Australian resource sector interventions that reduce workplace bullying.

## Background

The resources sector contributes substantially to the Australian economy [1]. Most Australian resource projects take place either in isolated geographic regions or off-shore oil and gas reservoirs. These regions generally have small residential populations and lack the infrastructure and services to draw people to the area, which results in insufficient skilled local labour [2]. Also, in the initial brief but labor intensive construction phase of a resource project, it is infeasible to permanently relocate workers to the area, since their positions become redundant once construction has concluded [3].

In light of the aforementioned employment difficulties, Australian resource companies have increasingly drawn on the use of Fly-In-Fly-Out (FIFO) or Drive-in-Drive-Out (DIDO) staff to satisfy their labor requirements [2] (note that from hereafter, FIFO will be used to refer collectively to both FIFO or DIDO employees). FIFO employment has been defined as “all employment in which the work is so isolated from the workers’ homes that food and accommodation are provided for them at the work site, and rosters are established whereby employees spend a fixed number of days at the site, followed by a fixed number of days at home” [4].

Recent Australian government reports have raised concerns about Australian FIFO workers’ wellbeing, especially their mental health [3, 5, 6]. Bullying has been proposed to be a key workplace issue that impacts on the mental health of Australian FIFO employees [7]. Empirical research on bullying in the Australian resource sector has not been undertaken. However, studies of bullying in other types of workplaces could provide insight into the factors that may influence bullying among FIFO employees.

Workplace bullying involves instances in which an employee continually encounters aggressive and negative behaviour, which has an intimidating, humiliating, punishing, or frightening effect on the individual experiencing such behaviour [8]. These acts when they occur in isolation can be characterised as uncivil workplace behaviour [9]. Uncivil behaviour, though, evolves into workplace bullying when it manifests repetitively over a prolonged period of time [10].

Workplace bullying may take the form of either direct or indirect acts of negative or aggressive behaviour [11]. For example, direct acts could involve verbal abuse and public humiliation, whereas indirect acts may consist of gossiping and rumours [11]. Workplace bullying can be further differentiated in terms of person-related behaviour and workplace-related behaviour [12]. Person-related bullying may comprise behaviour such as slander or social isolation, whereas workplace-related bullying may manifest in acts such as ongoing criticism of an individual or being assigned inappropriate tasks [12]. Another distinction worth considering in the operationalisation of workplace bullying is the difference between subjective and objective experience of bullying [13]. Subjective bullying reflects the targeted individual’s perceived experience [13]. In contrast, objective bullying needs to be verified through observers [13]. It should be noted that subjective experience may be the most valid measure of workplace bullying, since it is the subjective experience that will result in physical or mental health problems [14].

The final integral aspect of workplace bullying involves an imbalance of power between the perpetrator and target of uncivil behaviour [15]. Negative or aggressive behaviour is not characterised as workplace bullying if it occurs between individuals of the same status [15]. Uncivil behaviour is only considered as workplace bullying when an power imbalance leaves the target unable to protect or defend themselves against further ongoing negative acts [15, 16].

Systematic reviews have established that estimates of the prevalence of workplace bullying vary according to the manner in which bullying is defined. In studies where a given definition is provided, and in which individual’s self-label themselves as targets of bullying, the prevalence of bullying stands at 11.3% [17]. Studies that use behavioral self-report measures find that prevalence of bullying is 14.8%[17]. And in studies in which individual’s self-identify as targets of bullying without a provided definition, the prevalence is 18.1% [17]. Regardless of the method used to measure bullying, the meta-analytic results indicate that bullying is a substantial problem in contemporary workplaces.

Numerous antecedents of workplace bullying have been identified, which have been broadly conceptualised as either individual level, group level or organisational factors. In terms of the individual level, studies have shown conflicted findings about the association between personality profiles and workplace bullying [18]. However, while mixed results have been reported for many personality traits such as conscientiousness, extraversion and agreeableness [19–21], higher levels of neuroticism have been consistently observed in targets of bullying [18]. Also, preexisting mental health problems have been predictive of subsequent exposure to bullying [22, 23]. Finally, individuals with higher levels of negative affect, or lower self-esteem, more frequently experience bullying [19–21, 24]. But bullying itself may lead to low self-esteem and high negative affect, which casts doubt over whether these personality traits are antecedents of workplace bullying [25].

Individual level demographic factors have tended to be inconsistently associated with workplace bullying. For instance, one study failed to identify an association between age and bullying [26], yet another study reported that older employees were more likely to experience bullying in comparison to younger employees [27]. In addition, some studies have shown that females are more likely to experience bullying than males [28, 29], but other studies have demonstrated marginal or no differences between genders [8, 27]. Finally, males tend to only experience bullying from other males, whereas females are targeted by both genders but more often by other females [30].

Studies have identified several individual level factors that lead to the perpetuation of bullying. First, males are more likely to enact bullying than females [27, 31]. Second, individuals are more likely to engage in bullying when they are employed in positions with low job autonomy and high workloads [32]. Third, targets of bullying are more likely to be perpetrators of bullying [32, 33]. Finally, employees who experience job insecurity are more likely to enact workplace bullying [34].

At the individual level, exposure to workplace bullying leads to the development of health complaints, which can be broadly categorised as either mental health problems or somatic issues. Common mental health problems that result from workplace bullying include depression, anxiety, worry, distress, and suicidal ideation [35, 36]. However, when considering whether bullying has led to mental health problems, it should be noted that the relationship is bi-directional [22, 23]. Hence, pre-existing mental health problems increase the likelihood of an individual becoming a target of bullying [22, 23]. Somatic complaints that frequently occur in people who have experienced workplace bullying can involve headaches, sleep disorders, neck pain, fibromyalgia, and decreased general physical health [37–39]. The overall impact of bullying on health complaints was demonstrated through a systematic review which concluded that individuals who experienced workplace bullying were about twice as likely to report mental health or somatic problems [40]. Finally, in terms of individual level work-related outcomes, bullying results in an increased likelihood of absenteeism [41] and intent to leave [42], and lower levels of job satisfaction [43].

Several studies have also identified group-level antecedents of workplace bullying. Employees tend to become more aggressive after observing aggressive behaviour by other colleagues [44, 45]. Such instances tend to lead to employees taking the side of either the perpetrator or target but witnesses of bullying typically support the perpetrator in order to avoid becoming a subsequent target [46] This in turn can reinforce negative group norms that are accepting of bullying behaviour and encourage the perpetrator to engage in further acts of incivility [47–49].

Other antecedents of bullying at the group level include situational factors. For instance, higher levels of bullying within groups has been associated with higher levels of task conflict i.e. when disagreements between group members about the content of decisions owe to differences in viewpoints and opinions [50]. Another factor involves low levels of communication openness in a group, which results in an increased likelihood of bullying between group members [50]. Finally, self-managed teams exhibit higher levels of bullying, whereas lower levels of team autonomy have been associated with reduced instance of bullying behaviour [51]. This may be due to the fact that self-managed teams experience increased pressure and stress as a result of task independence and peer monitoring, which can promote opportunities to exert social power and status through bullying [51].

Group level consequences of workplace bullying include an increased likelihood of targets and perpetrators becoming isolated within the team [52]. The occurrence of bullying in groups also has a detrimental impact of perceptions of team success [52]. And as aforementioned, bullying in groups tends to result in further instances of bullying [48, 53].

At the organisational level, there are several factors that have been shown to be antecedents of workplace bullying. These factors can be broadly categorised as: 1) management and leadership style; 2) organisational culture; 3) situational factors; and 4) organisational policies.

Particular leadership and management styles have been associated with an abuse of power. For example, weak leadership may promote bullying through a failure to resolve conflicts [54]. Also, weak leadership groups may be less likely to intercede in instances of bullying, which may result in the perpetrator believing that they won’t be caught or punished [29]. Hence, the perpetrator may be more likely to enact further bullying [26, 29, 41]. Another leadership and management issue is the use of an authoritarian approach. Individuals who experience workplace bullying often report that their organisation adopts an authoritarian form of management [26]. Authoritarian management styles may promote a sense of fear, which incidentally can lead to supervisors using bullying as an instrument to exercise authority [55].

Several aspects of an organisation’s culture can be antecedents of workplace bullying [43]. Strong power imbalances can lead to circumstances that facilitate and institutionalise workplace bullying [29]. Competitive workplace environments also have been shown to promote bullying behaviour [19, 29]. In some organisations bullying has been viewed as a means of improving performance. Similarly, toughness may be encouraged in particular organisational cultures, which can aggravate bullying behaviour [29, 56]. Finally, it is worth noting that there may be a bi-directional relationship between individual characteristics and organisational culture [57]. While the culture within an organisation can promote bullying, employing an aggressive individual on the other hand can lead to the development of an organisational culture that endorses workplace bullying [12, 55].

Another antecedent of workplace bullying is organisational policies. Such policies provide guidance about behaviors in an organisation that are acceptable and unacceptable [58]. Employees may be unable raise attention about workplace bullying unless there is an explicit policy about it [58]. Moreover, the lack of a bullying policy can lead to employees believing that their organisation tolerates bullying [29].

Situational factors can also be an antecedent of workplace bullying. In particular, reward structures [29], work organisation [55], and organisational change or restructures [59] can promote workplace bullying. For instance, organisational restructures or change can cause job insecurity, which predicts workplace bullying [34, 59]. Types of work organisation like minor, repetitive duties may be frustrating and may also be associated with workplace bullying [10, 55]. Finally, reward structures that offer incentives for employees who perform in a superior manner to co-workers can result in bullying through behaviour that weakens competition [29].

The organisational level consequences of workplace bullying are similar in some respects to the consequences of individual level bullying. These similarities include absenteeism [36] and intent to leave [37], and lower levels of job satisfaction [38]. Other organisational consequences include reduced productivity [41, 60], litigation impacts such as reputational costs [61], and industrial action [61].

Anecdotal reports indicate that workplace bullying is an issue of particular concern for Australian FIFO workers. As an example, in 2014 the Department of Mines and Petroleum placed recruitment notices for workplace trainers, who would assist their inspectors in addressing increased bullying complaints. The Construction, Forestry, Mining, and Energy Union’s Western Australian safety officer noted that “official complaints were just the tip of the iceberg”, and many complainants had lost their job [7]. In addition, Mates in Construction WA, a suicide prevention group with a wide FIFO client base, reports that workplace bullying accounts for about 15% of its case-management activities [62].

As detailed in this review of the literature, there are number of workplace factors that promote bullying and psychosocial distress, which may also potentially influence uncivil behaviour and health outcomes for FIFO workers. The following section provides further details about how these factors may be influenced by the specific context of FIFO workplaces. Age has been inconsistently associated with workplace bullying [32], and it would be worthwhile to determine whether particular age groups in FIFO workers experience higher or lower levels of bullying. In workplaces where one gender predominates, such as FIFO workplaces that are largely composed of male employees, the gender in the minority tends to experience higher levels of bullying [63]. Senior employees are more likely to be perpetrators of bullying, which may moderate the relationship between bullying and psychosocial distress among FIFO workers [51]. In contrast to most other workplaces, FIFO workers are more likely to work night shifts and high compression rosters, which have been associated with psychosocial distress and therefore are important to investigate for confounding effects [64, 65]. Another potential moderating factor is alcohol consumption, which has been associated with higher levels of psychosocial distress in FIFO workers [66]. Finally, it is timely to examine if the promotion of collaboration influences bullying in FIFO workplaces, given that communication openness and leadership styles have been shown to mitigate bullying in other workplaces [50, 54].

Despite the anecdotal reports of FIFO bullying, no prior studies have examined the issue empirically in a FIFO worker cohort, particularly in terms of the manner in which bullying affects psychosocial distress. Hence, the aim of this study was to address the literature gap through: 1) establishing the prevalence of bullying in Australian FIFO workers in the resources sector; 2) identifying predictors of bullying in FIFO workers; 3) examining the association between bullying and clinical depression for FIFO workers; and 4) assessing the relationship between bullying and suicide risk in FIFO employees. We hypothesised that among FIFO workers higher levels of bullying would be positively associated with: female gender; younger age; lower educational levels; not in relationship; alcohol available at workplace; non-manager or non-supervisor roles; high compression rosters; working night shifts; failure of supervisors to promote collaboration; clinical depression; and suicide risk.

## Methods and materials

### Study design

A cross sectional survey design was used to undertake this study. The Murdoch University Human Research Ethics Committee provided ethical approval (Project number: 2014/039).

### Participants

The questionnaire captured details about whether prospective participants were FIFO, residential, or some “other” (open-ended question) type of employee in the resources sector. Prospective participants were eligible for inclusion in this study if they were adults, currently employed only as FIFO workers in the Australian resources sector. Recruitment notices and information letters were distributed at industry training seminars and site visits, and the survey was also promoted through Facebook. The information sheet notified prospective participants that completion of the online survey was voluntary and their identities would remain anonymous. Return of the survey questionnaire was used to indicate informed consent.

### Measures

The Negative Acts Questionnaire-Revised (NAQ-R) was used to assess workplace bullying [14]. This measure assesses subjective perceptions of bullying, but does not explicitly enquire about bullying [14]. The NAQ-R instead captures details using behavioural terms without directly mentioning the terms “bullying” or “harassment”, which is considered to result in more objective estimates than the self-labelling of bullying behaviour [14]. These behavioural terms have been grouped into the following three factors: personal bullying, work-related bullying, and physically intimidating types of bullying [14]. Although the NAQ-R contains three factors, an analysis has shown that all of the items within these factors load onto one factor that has a high Cronbach alpha value of 0.90, which supports its use as a single factor measure [14]. The NAQ-R has been shown to be a valid and reliable measures in diverse workplace settings [36, 67–70]. It consists of 22 items, each of which is scored on a five point Likert scale ranging from 1–5. All items are summed to produce an overall scale score, which is categorised as: < 33 equals “no bullying”; 33 ≤ 45 equals “occasional bullying”; and > 45 equals “severe bullying”. Prior research has established the sensitivity and specificity of these cut-points [71].

The Beck Depression Inventory-II (BDI-II) was used to establish clinical depression [72]. Numerous psychometric studies have provided evidence for its validity and reliability [72–74]. The BDI-II contains 21 items, scored from 0–3, and summed to result in an overall score. A score of 21 or more indicates clinical depression [72]. The Cronbach’s alpha for the BDI-II derived from the responses of this study’s cohort was 0.90, which demonstrates a high level of internal consistency.

Hopelessness was measured with the Beck Hopelessness Scale (BHS) [75]. It has been shown to be significantly superior in identifying suicidal intention and behaviour than the BDI-II [76, 77]. Extensive prior research has consolidated the BHS’s psychometric properties [69, 78]. This instrument comprises 20 items, of which some are reverse coded [75]. Each item is assigned a value of either zero or one, with pessimistic responses summed to result in an overall score ranging between 0–20 [4]. Scores ≥ 9 suggest elevated suicide risk[78, 79]. The Cronbach’s alpha for the BHS derived from the responses of this study’s cohort was 0.92, which demonstrates a high level of internal consistency.

Finally, the questionnaire also captured details about the following demographic and workplace variables: age; gender; relationship status; educational attainment; employment level; whether the supervisor promotes collaboration; workplace alcohol availability; and drug use.

### Data analysis

All data were analysed in SPSS v.24 and reported descriptively. Backward Logistic regression was used to examine predictors of bullying, and the association between bullying, suicide risk, and clinical depression. The dependent variables bullying, suicide risk and clinical depression were entered into separate multivariate logistic regression models. For the predictive bullying model, the dependent variable was derived through the dichotomising the NAQ- R overall score as < 33 equals “no bullying” and ≥ 33 equals “bullying”. This dichotomised variable was also entered as the independent variable into the models examining clinical depression and suicide risk. The dependent variables of “clinical depression” and “suicide risk” were derived through the dichotomising the BDI-II and BHS scores at their clinical cut points. The following variables were entered as predictors in the bullying model, and as confounders in the clinical depression and suicide risk models: age (below median value = 0; above median value = 1; gender (male = 0; female = 1); education (high school or below = 0; Technical And Further Education = 1; Bachelor degree or above = 3); relationship status (single, divorced, separated, or widowed = 0; current relationship, engaged, or married = 1); supervisor doesn’t promote collaboration (no = 0; yes = 1); roster type (non-high compression = 0; high compression = 1) employment level non-managerial or non-supervisory role = 0; managerial or supervisory role = 1); and alcohol workplace availability (yes = 0; no = 1).

## Results

### Respondent characteristics

Questionnaires were received from 580 respondents, some of which were incomplete (See Table 1 for number of responses per demographic variable). The survey response rate was indeterminable, as some study invitation notices were distributed through Facebook and the number of potential participants who accessed them was unknown.

**Table 1:** Demographic characteristics.

Variable (number)		Mean (Standard Deviation)
Age (n = 576)		35.5 (9.1)
		Proportion
Gender (n = 574)	Male	76.3
Education (n = 577)	High school or below	41.8
	Technical And Further Education	42.6
	Bachelor degree or higher	15.6
Relationship status (n = 506)	Married/engaged/or have current partner	73.7
Employment level (n = 524)	Supervisor/manager	20.0
Supervisor promotes collaboration (n = 362)	Yes	39.8
Workplace alcohol availability (n = 356)	Yes	11.2
Work daytime shift (n = 515)	Yes	55.7
High Compression Roster (n = 408)	yes	19.0
Industry (n = 507)	Mining	18.6
	Oil and Gas	7.0
	Multiple Industries	12.4
	Construction	9.3
	Services	14.7
	Transport	5.4
	Hospitality	7.0
	Medical/Health	12.4
	Education	9.3
	Other	2.3
State (n = 491)	Australian Capital Territory	0.0
	New South Wales	5.1
	Northern Territory	0.2
	Queensland	35.6
	South Australia	5.9
	Victoria	12.4
	Western Australia	57.4

The demographic characteristics of the respondents are displayed in Table 1. The respondents average age was 35.5 (SD = 9.1) years. Most respondents were male (76.3%). In terms of education, about four in ten held either high school (41.8%) or Technical And Further Education qualifications (42.6%), and the remainder held Bachelor degree or post-graduate qualifications (15.6%). Almost three-quarters of the respondents were currently in a relationship (73.7%). One in five held a supervisory or managerial role (20.0%). Less than half of the respondents stated that their supervisor successfully promoted collaboration between colleagues (39.8%). Almost one in five were on a high compression roster (19.0%), and over half worked daytime shifts (55.7%). Finally, about one in ten were employed in workplaces where alcohol was available (11.2%).

### Prevalence of bullying in FIFO workers

The mean NAQ-R was 39.2 (SD = 16.2). Examining the cut-points for the NAQ-R showed that 28.6% of the FIFO workers experienced occasional bullying and 27.1% reported severe bullying.

### Antecedents of bullying

Table 2 present the results of the logistic regression analysis examining antecedents of bullying. Being above the median age was associated with a fifty per cent reduction in the likelihood of experiencing bullying (OR = 0.51; 95% CI = 0.31–0.83). In addition, the failure of supervisors to promote collaboration was associated with employees being three times more likely to report bullying (OR = 3.04; 95% CI = 1.84–5.04)

**Table 2:** Results of logistic regression model examining antecedents of bullying.

	B	S.E	p Value	Odds Ratio	95% CI
Age	-0.68	0.25	0.006	0.51	0.31–0.83
Gender	-0.18	0.31	0.56	0.83	0.45–1.54
Roster Type	-0.24	0.26	0.36	0.79	0.48–1.31
Employment Level	0.17	0.30	0.58	1.18	0.65–2.14
Night Shift	0.14	0.21	0.51	1.1	0.77–1.71
Supervisor Doesn’t Promote Collaboration	1.11	0.26	0.0001	3.04	1.84–5.04
Relationship Status	0.33	0.29	0.25	1.39	0.79–2.45
Alcohol Availability	-0.32	0.37	0.39	0.73	0.35–1.51
Technical And Further Education	0.24	0.27	0.37	1.27	0.75–2.16
Tertiary Education	-0.07	0.37	0.85	0.93	0.45–1.93

### Prevalence of depression and hopelessness in FIFO workers

The BDI mean value was 16.3 (SD = 11.3). Overall, about one-third of FIFO workers (32.3%) reported moderate or more severe depression. The BHS mean score was 5.8 (SD = 5.4). Based on the established BHS cut-point, about one-quarter of FIFO workers were at elevated risk of suicide (26.7%).

### Association between bullying, clinical depression, and suicide risk in FIFO workers

Full details for the logistic regression analyses are displayed in Tables 3 and 4. The results demonstrated that bullying (OR = 2.38; 95% CI = 1.40–4.05) was associated with participants being almost two and a half times more likely to report clinical depression. Bullying was also associated with an almost threefold increase in suicide risk (OR = 2.70; 95% CI = 1.53–4.76). The Nagelkerke R^2^ for the logistic regression models examining clinical depression and suicide risk respectively were 0.09 and 0.11, which indicates that the variables within these models accounted for 9.0% of the variation in clinical depression and 11.0% of the variation in suicide risk. The values for the Chi-Square goodness of fit test for the logistic regression models examining clinical depression and suicide risk respectively were 0.0001 and 0.005, which indicated that there were significant differences between the observed and expected frequencies.

**Table 3:** Results of logistic regression model examining association between bullying and suicide risk.

	B	SE	p Value	Odds Ratio	95% CI
Bullying	0.99	0.29	0.001	2.70	1.53–4.76
Age	0.37	0.28	0.18	1.5	0.84–2.50
Gender	-0.41	0.35	0.24	0.67	0.34–1.30
Roster Type	-0.35	0.38	0.34	0.68	0.33–1.48
Employment Level	0.24	0.36	0.49	1.28	0.63–2.60
Night Shift	0.48	0.27	0.08	1.62	0.95–1.94
Supervisor Doesn’t Promote Collaboration	0.31	0.28	0.26	1.36	0.08–2.36
Relationship Status	0.37	0.29	0.21	1.44	0.82–2.54
Alcohol Availability	0.07	0.43	0.87	0.03	0.46–2.50
Technical And Further Education	-0.63	0.30	0.03	0.53	0.30–0.95
Tertiary Education	-0.09	0.38	0.81	0.92	0.43–1.94

**Table 4:** Results of logistic regression model examining association between bullying and clinical depression.

	B	SE	p Value	Odds Ratio	95% CI
Bullying	1.01	0.29	0.0001	2.38	1.40–4.05
Age	-0.25	0.27	0.35	0.78	0.46–1.32
Gender	0.01	0.34	0.98	0.98	0.48–2.00
Roster Type	-0.02	0.48	0.96	1.01	0.52–2.00
Night Shift	0.06	0.29	0.83	1.06	0.61–1.87
Employment Level	-0.13	0.33	0.69	0.88	0.46–1.67
Supervisor Doesn’t Promote Collaboration	0.87	0.27	0.001	2.39	1.41–4.06
Relationship Status	0.19	0.30	0.52	1.21	0.68–2.16
Alcohol Availability	-0.06	0.43	0.89	0.95	0.41–2.21
Technical And Further Education	-0.27	0.30	0.38	0.75	0.44–1.37
Tertiary Education	-0.21	0.40	0.59	0.78	0.36–1.73

## Discussion

Over half of the respondents in this study experienced bullying, which highlights that this issue reaches alarming levels in the Australian resource workplace. Also of concern in this cohort were the associations between bullying and elevated rates of clinical depression and suicide risk. Our findings indicate that supervisors can contribute importantly to the mitigation of bullying, as workers whose supervisors did not promote collaboration were almost three times more likely to experience bullying.

The estimated prevalence of bullying identified in this study was about four times higher than the rate reported in a systematic review of workplace bullying that pooled prevalence estimates from 34 studies that had used similar behavioural self-report measures [17]. Such disparity in prevalence rates suggests that there are particular workplace factors in the Australian resource sector that exacerbate bullying behaviour.

The high rate of bullying in FIFO workers observed in this study may have owed to the preponderance of male workers, who are more likely to enact bullying [30]. Similarly, it has been proposed that bullying is higher in male dominated industries due to a generally higher level of aggression among males [80].To some extent, though, these notions are countered by the fact that studies of nursing, a profession predominated by women employees, report similar rates of bullying to the rate observed in this study [81]. Hence, it may the case that bullying rates are high in professions where a single gender predominates. But other studies have shown that in workplaces where one gender significantly prevails, it is the gender in the minority that experiences elevated levels of bullying [63, 82]. However, findings from other studies suggest the type of occupation may be more important than distribution of gender [16]. Given the mixed findings about the role of gender in uncivil workplace behaviour, further studies are warranted to examine this issue among FIFO employees.

The FIFO workplace environment, which typically is similar to a total institution, might be also be associated with the high rate of bullying found in this study [83]. Most Australian resource workplaces in isolated regions consist of camps, which often contain hundreds of identical dongas (temporary, typically transportable building with a single room) structured in grid format, enclosed by barbed wire fences. Not only do most FIFO workplaces resemble prison camps, they also operate in an institutionalized manner. FIFO workers note that excessive regulations and highly regimented structures strip them of control over their work and personal time, which is a significant source of distress that is further compounded by separation from loved ones.[84] In the case of total institutions, such loss of autonomy and separation from loved ones results in substantial psychological trauma^24^. In self-preservation, people create subcultures based on ultra-masculinity and dominance, and engage in maladaptive behavior like bullying, as a means to exert control over their situation [83].

The highly regulated nature of FIFO workplaces may also reflect an authoritarian management style, which is a predictor of bullying. Authoritarian management approaches are thought to promote bullying in two primary ways. First, authoritarianism may create a fearful atmosphere, in which employees may believe that criticism is not tolerated and complaints will not be acted upon [26]. Second, authoritarian management leads to abusive supervision, whereby supervisors use an aggressive style to influence control over employees [85].

Our estimation of the one in three rate of clinical depression in FIFO workers in the Australian resource sector was considerably higher than the rate of one in twenty-five found in the general Australian population [86]. In addition our findings demonstrated FIFO workers were at greater risk of suicide than the general population, as about one in four reported increased risk whereas one in six in the general population experience increased risk [87]. Bullying contributed significantly to depression and suicide risk, as indicated by the odds ratios that demonstrated around a two and a half increase in both depression and suicide risk in participants who experienced bullying. These findings highlight the need to implement interventions that reduce bullying in resource workplaces.

As a starting point to reduce bullying in the FIFO sector, employees in leadership positions should take note of the issue and proactively adopt management styles that reduce bullying [26, 29, 49]. Organisational policies should also be examined to ensure that they explicitly state that bullying will not be tolerated and detail how instances of bullying will be managed [29, 58]. Employee incentives could be reviewed to ensure that they are not contributing to the perpetration of bullying [20, 29]. Finally, employees throughout all levels of the organisation could be educated about what constitutes bullying and how it could be mitigated.

The evidence base for interventions that reduce workplace bullying has methodological shortcomings but nonetheless provides some useful guidance [22, 79]. Prevention strategies fall into several types: primary, secondary, and tertiary. Primary strategies mainly aim to mitigate the incidence of bullying through contextual alterations and educational workshops or employee training. Secondary intervention focus on providing individuals with coping strategies to manage bullying if it occurs. And tertiary strategies deliver support and assistance to bullied employees in order to resolve its effects. Most studies in the prevention of workplace bullying have shown modest reductions, and no one type of prevention intervention is particularly superior to other types [79]. However, all studies that demonstrated reductions in bullying were cross-sectional, and the only available controlled study found that the intervention had no significant effect on bullying [79]. Hence, further controlled studies are warranted to consolidate the evidence for the effectiveness of interventions in mitigating workplace bullying[22].

The findings of recent reviews about interventions to mitigate workplace bullying do not appear to have examined how it could be reduced by managers or supervisors enhancing the manner in which colleagues work together [22, 79, 88]. But our results suggest that workplace bullying could be reduced if supervisors promoted collaboration. It may be the case that collaboration leads to an open atmosphere in which bullying issues may be more readily identified, resulting in early resolution of uncivil behaviour. However, we did not examine the processes that underpinned the promotion of collaboration. As such, additional studies are required to develop a more nuanced understanding of how workplace bullying could be mitigated through the promotion of collegial collaboration.

### Limitations

The number of respondents in this study was substantial, but it is unclear if their views were representative in general of FIFO workers in the Australian resource sector. However, the demographic characteristics of our respondents were consistent with details reported in a parliamentary enquiry into the Australian resource sector, which provides tentative support for the generalisability for this study’s findings [5]. It should be noted, though, that the cross-sectional study design precluded identifying whether the participants’ high level of depression was present before entering employment in the resources sector. Moreover, given the cross-sectional study design, it is uncertain if the association we identified between depression and bullying was causative in nature. Therefore, longitudinal studies are warranted to verify our findings, which is necessary to better understand the relationship between bullying and psychosocial distress in FIFO workers, particularly as studies in other populations have shown that this association is bi-directional [22]. Another issue that should be taken into consideration is that depressed individuals may be more likely to participate in surveys, and that the anonymous nature of our survey may have reduced stigma about detailing mental illness. However, studies have shown conflicted results about the influence of data collection methods on revealing sensitive material, and it is unclear whether anonymous surveys increase reporting rates for psychosocial distress [76].

## Conclusion

Our findings show that the incidence of bullying and suicide risk in Australian FIFO workers has reached alarming proportions. Moreover, bullying was significantly associated with clinical depression and suicide risk, the prevalence for both of which was substantially higher than the rate in the Australian general population. The results of this study highlight the need to implement in the Australian resource sector interventions that reduce workplace bullying. Individuals in leadership positions should review their management style and organisational policies to ensure that they do not contribute to workplace bullying. Interventions should also be delivered to teams to ensure that the manner in which they interact does not perpetuate bullying, and it could be especially worthwhile to deliver interventions that promote individual resilience to enable targets to better cope with the impacts of bullying behaviour. Finally, our findings indicate that workplace bullying accounted for a relatively small proportion of the variance in clinical depression and suicide risk in Australian FIFO workers. Given the high levels of clinical depression and suicide risk in this cohort, it is important to undertake further studies to more comprehensively understand factors that contribute to such psychosocial distress. Identification of these factors would enable employers to deliver targeted approaches that may enhance the reduction of clinical depression and suicide risk in FIFO workers.
